# Hepatitis C virus fitness can influence the extent of infection-mediated epigenetic modifications in the host cells

**DOI:** 10.3389/fcimb.2023.1057082

**Published:** 2023-03-13

**Authors:** Carlos García-Crespo, Irene Francisco-Recuero, Isabel Gallego, Marina Camblor-Murube, María Eugenia Soria, Ana López-López, Ana Isabel de Ávila, Antonio Madejón, Javier García-Samaniego, Esteban Domingo, Aurora Sánchez-Pacheco, Celia Perales

**Affiliations:** ^1^ Department of Interactions with the Environment, Centro de Biología Molecular “Severo Ochoa” (CSIC-UAM), Consejo Superior de Investigaciones Científicas (CSIC), Campus de Cantoblanco, Madrid, Spain; ^2^ Centro de Investigación Biomédica en Red de Enfermedades Hepáticas y Digestivas (CIBERehd), Instituto de Salud Carlos III, Madrid, Spain; ^3^ Department de Biochemistry, UAM, Instituto de Investigaciones Biomédicas Alberto Sols, CSIC-UAM, Madrid, Spain; ^4^ Department of Clinical Microbiology, IIS-Fundación Jiménez Díaz, UAM, Madrid, Spain; ^5^ Hepatology Unit Hospital Universitario La Paz/Carlos III, Instituto de Investigación Sanitaria “La Paz”, Madrid, Spain; ^6^ Department of Molecular and Cell Biology, Centro Nacional de Biotecnología (CNB-CSIC), Consejo Superior de Investigaciones Científicas (CSIC), Madrid, Spain

**Keywords:** virus-host interaction, histone modification, viral quasispecies, hepatocellular carcinoma, viral fitness, hepatitis C virus, aurora kinase B

## Abstract

**Introduction:**

Cellular epigenetic modifications occur in the course of viral infections. We previously documented that hepatitis C virus (HCV) infection of human hepatoma Huh-7.5 cells results in a core protein-mediated decrease of Aurora kinase B (AURKB) activity and phosphorylation of Serine 10 in histone H3 (H3Ser10ph) levels, with an affectation of inflammatory pathways. The possible role of HCV fitness in infection-derived cellular epigenetic modifications is not known.

**Methods:**

Here we approach this question using HCV populations that display a 2.3-fold increase in general fitness (infectious progeny production), and up to 45-fold increase of the exponential phase of intracellular viral growth rate, relative to the parental HCV population.

**Results:**

We show that infection resulted in a HCV fitness-dependent, average decrease of the levels of H3Ser10ph, AURKB, and histone H4 tri-methylated at Lysine 20 (H4K20m3) in the infected cell population. Remarkably, the decrease of H4K20m3, which is a hallmark of cellular transformation, was significant upon infection with high fitness HCV but not upon infection with basal fitness virus.

**Discussion:**

Here we propose two mechanisms ─which are not mutually exclusive─ to explain the effect of high viral fitness: an early advance in the number of infected cells, or larger number of replicating RNA molecules per cell. The implications of introducing HCV fitness as an influence in virus-host interactions, and for the course of liver disease, are warranted. Emphasis is made in the possibility that HCV-mediated hepatocellular carcinoma may be favoured by prolonged HCV infection of a human liver, a situation in which viral fitness is likely to increase.

## Introduction

Epigenetic modifications modulate gene expression programs, with implications that have been extensively studied for the phenotypic profile of cancer cells, and their functional heterogeneity [reviewed in (Ilango et al., 2020; Ramon et al., 2020)]. Host cell epigenetic signatures are also altered during infection by DNA and RNA viruses, with consequences for viral persistence and latency (Milavetz and Balakrishnan, 2015; Lynch et al., 2019). Hepatitis C virus (HCV) participates of the double branch of epigenetic implications since they may affect the course of the infection itself, and the development of post-infection sequels such as hepatocellular carcinoma (HCC), often linked to progression of liver cirrhosis. Epigenetic changes that involve aberrant methylation of genes and post-transcriptional histone modifications occur frequently, and some of them are being exploited for the development of molecular diagnostic signatures for HCC (Lin et al., 2015; Zheng et al., 2019).

Several studies have addressed the interference of HCV-encoded proteins exerted on epigenetic pathways, in the course of the infection (Hung et al., 2014). Our previous investigation of HCV-induced histone modifications at early stages of HCV infection revealed inhibition of phosphorylation of Ser10 in histone H3 (H3Ser10ph), associated with a decrease of Aurora Kinase B (AURKB) activity, mediated by a direct interaction between HCV core protein and AURKB. In addition, we showed that AURKB inhibition had an effect on transcription of genes related to inflammatory pathways such as *NF-кB* and *COX-2* (Madejón et al., 2015). Aurora B is involved in chromosome segregation, spindle-checkpoint and cytokinesis, and alteration of each of these mitotic processes could induce aneuploidy, one of main features of cancer cells (Hegyi and Mehes, 2012; Chieffi, 2018). Chronic HCV infection also induces genome-wide changes in H3K27 acetylation, dependent on the liver fibrosis stage (Hamdane et al., 2019). Moreover, significant HCV infection-associated changes in active chromatin markers H3K4Me3 and H3K9Ac and silent chromatin marker H3K9Me3 seem to be associated with alteration of expression of genes involved in HCC development (Perez et al., 2019). Therefore, several lines of evidence suggest that epigenetic modifications as a result of chronic HCV infection may contribute to cancer risk (Mann, 2014; Madejón et al., 2015; Lohmann and Bartenschlager, 2019; Perez et al., 2019).

In the studies on the effect of HCV infection on host epigenetic modifications, the influence of viral replicative fitness is unknown. Yet, viral fitness, defined as the capacity of a viral population to produce infectious progeny, can have profound effects in the virus-host relationship, infection outcome, and response to antiviral treatment [reviewed in (Domingo and Holland, 1997; Quiñones-Mateu and Arts, 2006; Wargo and Kurath, 2011; Domingo et al., 2019; Domingo et al., 2020)]. Complex HCV quasispecies distributions evolve in infected patients (Farci, 2011; Quarleri and Oubina, 2016) with multiple implications for viral pathogenesis (Yan et al., 2008; Domingo et al., 2012).

**Figure 1:**
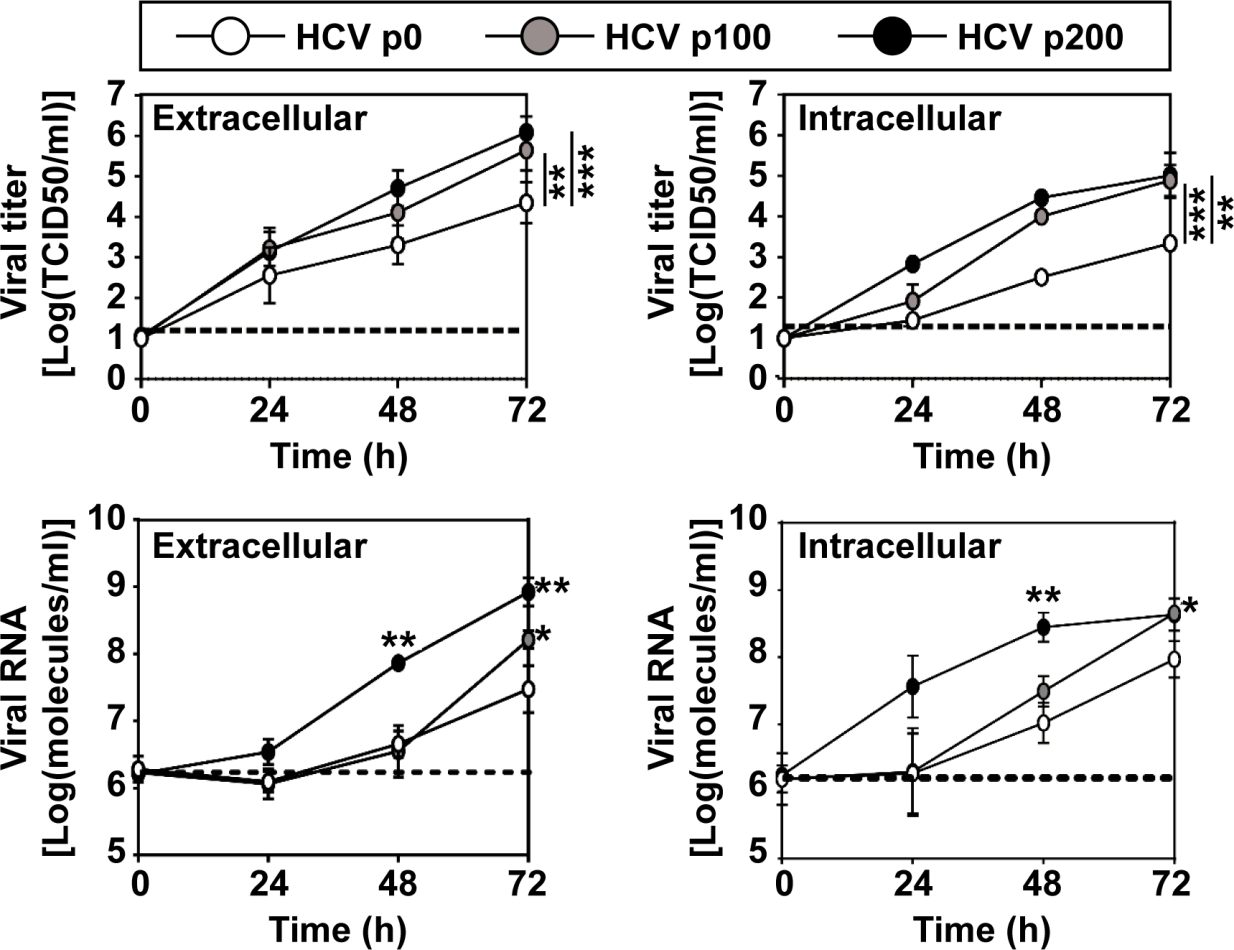
Kinetics of viral progeny production by HCV p0, HCV p100 and HCV p200. Huh 7.5 cells were either mock-infected or infected with the indicated virus (top box) at a MOI of 0.03 TCID_50_/cell (4 x 10^3^ Huh-7.5 cells infected with 1.2 x 10^4^ TCID_50_ of virus). Extracellular and intracellular infectivity and viral RNA were quantified. Results are the average of three independent experiments (biological triplicate). Data were transformed to the logarithmic values as indicated in ordinate. The statistical significance of the differences was calculated using the ANCOVA test (*p < 0.05; **p < 0.01; ***p < 0.001). Data on viral titers (upper two panels), have been previously published in (Moreno et al., 2017), and are included here to complement information on HCV fitness.

Most studies on HCV population dynamics have not considered viral fitness, presumably due to difficulties for its quantification *in vivo*. Prolonged replication of a clonal population of HCV (termed HCV p0, obtained by transcription of plasmid Jc1FLAG2(p7-nsGLuc2A) (Marukian et al., 2008; Perales et al., 2013), resulted in populations HCV p100 and HCV p200 (which are HCV p0 passaged 100 and 200 times, respectively, in Huh-7.5 cells) that exhibited significant increases in viral replication, calculated relative to HCV p0 (Moreno et al., 2017) (**Figure 1**). Taking HCV p0 as reference for fitness measurements (arbitrarily assigned a fitness value of 1) in growth-competition experiments in Huh-7.5 cells, HCV p100 and HCV p200 attained a fitness value of 2.3 (Sheldon et al., 2014; Moreno et al., 2017).

In serial passages in Huh-7.5 cells, HCV p100 and HCV p200 displayed a 1.8-fold and 2.8-fold, respectively, higher infectious progeny yield per passage than HCV p0, while the maximum extracellular infectivity level was 1.17-fold higher for HCV p100 and HCV p200 that for HCV p0, over a broad range of multiplicity of infection (MOI) (Moreno et al., 2017). Thus, while the relative fitness of HCV p100 and HCV p200 was the same according to growth-competition assays for fitness measurement (Domingo and Holland, 1997), the exponential intracellular growth rate was 2.6-fold larger for HCV p200 than for HCV p100 (Moreno et al., 2017) (**Figure 1**).

Fitness gain of HCV resulted in genetically and phenotypically heterogeneous population displaying broad mutant spectra (Domingo et al., 2020; Gallego et al., 2020; Delgado et al., 2021). Attainment of high fitness was related to mutant spectrum complexity, itself previously identified as a determinant of disease progression and response to treatment of HCV infections *in vivo* (Farci et al., 2000; Farci, 2011). In the cell culture system, high HCV fitness confers resistance to antiviral agents used in therapy (Sheldon et al., 2014; Gallego et al., 2016). There is evidence that fitness-enhancing substitutions may be also involved in treatment failures *in vivo* (Wang et al., 2013; Soria et al., 2020). Directly relevant to the virus-host relationship was the observation that high fitness HCV p100 and HCV p200 enhanced the shut-off of host cell protein synthesis relative to HCV p0, *via* increased phosphorylation of protein kinase R, and protein synthesis initiation factor eIF2α (Perales et al., 2013; Moreno et al., 2017). Given the multiple consequences of HCV fitness on host interactions, it was relevant to examine the possible influence of HCV fitness on host cell epigenetic signatures. Here we present comparative results with HCV p0, HCV p100 and HCV p200 that suggest such an influence. Mechanisms by which HCV fitness may modify quantitatively or even condition epigenetic modification in the host cells are discussed. The results render replicative fitness a relevant parameter for the interpretation of epigenetic modifications by this viral pathogen.

## Materials and methods

### Cell, viruses and infection

Huh-7.5 reporter cells were used for standard infections while Huh-7.5 cells were used for titration of virus infectivity. The origin of Huh-7.5 and Huh-7.5 reporter cell lines, and procedures for cell growth in Dulbecco’s modification of Eagle’s medium (DMEM), have been previously described (Blight et al., 2002; Jones et al., 2010; Perales et al., 2013); cells were cultured at 37°C and 5% CO_2_.

The initial HCV was rescued from plasmid Jc1FLAG2(p7-nsGluc2A), and GNN from plasmid GNNFLAG2(p7nsGluc2A) (which carries a mutation in NS5B that renders the virus replication defective) and used as a negative infection control (Marukian et al., 2008). The preparation of the initial virus, HCV p0, has been previously described (Perales et al., 2013). HCV p100 and HCV p200 resulted from population HCV p0 passaged 100 and 200 times, respectively, in Huh-7.5 reporter cells, as described (Sheldon et al., 2014; Moreno et al., 2017). Mock-infected cells or GNN-infected cultures were included in parallel to ascertain the absence of contamination; no infectivity was detected in any of these control cultures. In all experiments, Huh-7.5 reporter cells were either mock-infected or infected with HCV p0, HCV p100 or HCV p200 (abbreviated as p0, p100 and p200, respectively) at an initial MOI of 0.03 TCID_50_/cell, and processed 72, 96 or 144 hours after HCV infection, unless indicated in the relevant figure. Under these conditions 80-90% of the cells were infected as determined by live imaging (Jones et al., 2010).

### Virus titration

Titration of infectious HCV was performed by serial dilution of cell culture supernatants and applying them to Huh-7.5 cell monolayers in 96-well plates (6,400 cells/well, seeded 16 h earlier). Three days after infection, the cells were washed with PBS, fixed with ice-cold methanol, and stained to detect NS5A using anti-NS5A monoclonal antibody 9E10, as described previously (Lindenbach et al., 2005; Perales et al., 2013). Virus titers are expressed as TCID_50_ per millilitre (Reed and Muench, 1938).

### Western blot assays

Western blot assays were performed as previously described (Tardaguila et al., 2011). Blots were developed with the following antibodies: mouse monoclonal anti-core (ref. Sc-69937, Santa Cruz Biotechnology), mouse monoclonal anti-NS5A (ref. Sc-65458, Santa Cruz Biotechnology), rabbit polyclonal anti-H3Ser10ph (ref. 06-570, Millipore), rabbit polyclonal anti-AURKB (ref. Ab2254, Abcam), mouse monoclonal anti-β-Actin (ref. SAB1305567, Sigma), mouse monoclonal anti-tubulin (ref. T8328, Sigma), rabbit polyclonal anti-H4K20Me3 (ref. 07-463, Millipore), rabbit polyclonal anti-H3 (ref. 06-755, Millipore), mouse monoclonal anti-GADPH (ref. #CB1001, CallBiochem). Depending on the antibody, 5–30 µg of total protein was used in Western blot assays. β-Actin, tubulin, H3 or GADPH levels were used as loading control in Western blot assays. The values are the average of duplicate or triplicate determinations.

### RNA extraction and quantification

Intracellular viral RNA was extracted from infected cells using the Qiagen RNeasy kit (Qiagen, Valencia, CA, USA), following the manufacturer’s instructions. Viral RNA from cell culture supernatants was extracted using the Qiagen QIAamp viral RNA mini kit (Qiagen, Valencia, CA, USA).

HCV RNA quantification was performed by real-time quantitative RT-PCR (qRT-PCR) using the Light Cycler RNA Master SYBR green I kit (Roche) (Lindenbach et al., 2005; Perales et al., 2013). The 5’ untranslated region (UTR) of the HCV genome was amplified using as oligonucleotide primers HCV-5UTR-F2 5’-TGAGGAACTACTGTCTTCACGCAGAAAG-3’ (sense orientation; the 5’ nucleotide corresponds to genomic residue 47; according to JFH-1, GenBank accession number #AB047639) and HCV-5UTR-R2 5’-TGCTCATGGTGCACGGTCTACGAG-3’ (antisense orientation; the 5’ nucleotide corresponds to genomic residue 347; according to JFH-1) (Perales et al., 2013). Quantification was relative to a standard curve obtained with known amounts of HCV RNA synthesized by *in vitro* transcription of plasmid GNNFLAG2(p7-nsGluc2A). Negative controls (without template RNA and RNA from mock-infected cells) were run in parallel with each amplification reaction to ascertain the absence of contamination with undesired templates.

Cellular mRNA quantification was performed by RNA retro-transcription (RT) and real-time quantitative (qPCR). Intracellular RNA was retro-transcribed using AMV reverse transcriptase (Promega) following manufacturer’s instructions, and quantified using the NZYSpeedy qPCR Probe Master Mix (NZYTech). Each value was normalized against the GAPDH gene, and expressed as relative RNA abundance compared to mock-infected cells. The oligonucleotide primers used to amplify AURKB were 5’- GGGCGTCCTCTGCCCAAAGGC-3´ (sense orientation), 5´-GCCTGGATTTCGATCTCTC-3´ (antisense orientation), which resulted in amplification of nucleotides 360 to 511 of the AURKB gene. The oligonucleotide primers used to amplify GADPH were 5´-ACACTGCATGCCATCACTGCC-3´ (sense orientation), 5´-GCCTGCTTCACCACCTTCTTG-3´ (antisense orientation), which resulted in amplification of nucleotides 717 to 982 of the GADPH gene.

### Statistics

Linear regression’s test was performed using software R version 3.6.3. Student’s t and Wilcoxon tests were performed using the SSC-Stat software (version 2.18; University of Reading, Reading, UK) and the IBM SPSS Statistic 19 software. The statistical significance of differences between groups was expressed by asterisks (*=p <0.05; **=p <0.01; ***=p <0.001).

## Results

### Alteration of histone H3 Ser10 phosphorylation by infection of human hepatoma cells with HCV of different fitness

**Figure 2:**
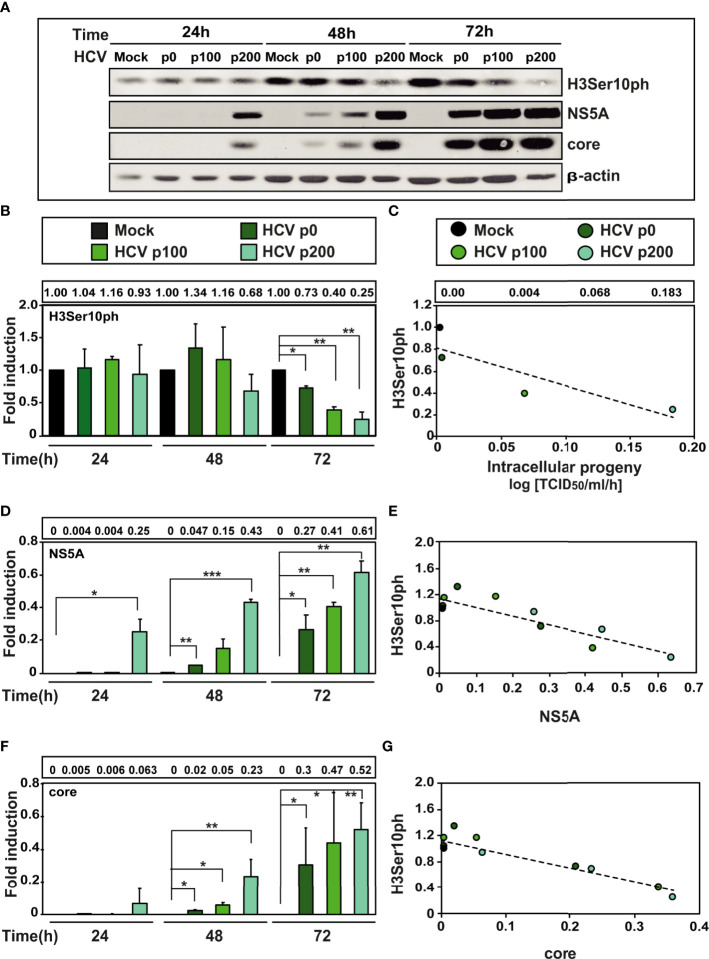
Effect of HCV fitness on the level of cellular protein H3Ser10ph and viral proteins NS5A and core. **(A)** Huh-7.5 reporter cells were either mock-infected or infected with HCV p0, HCV p100 or HCV p200 (abbreviated as p0, p100 and p200, respectively) at an initial MOI of 0.03 TCID_50_/cell; protein extracts were prepared at the indicated times post-infection. Bands are those visualized by Western blot assays of cellular protein H3ser10ph and viral proteins NS5A and core (with β-actin as loading control). **(B)** H3Ser10ph levels in mock-infected or HCV-infected cells expressed as the fold induction relative to the corresponding value for the mock-infected cells; the infecting HCV (code in upper box), and the time post-infection at which extracts were prepared are given in the abscissa; the numerical densitometry values (measured relative to the mock-infected sample, taken as 1) are indicated in the upper box, next to the panel. Asterisks indicate statistical significance as follows: *p<0.05; **p<0,01; ***p<0.00; unpaired t-test. The values are the result of three independent experiments (biological triplicate). **(C)** Correlation between the increase of intracellular viral titer (code of infecting virus in upper box) expressed as log. 10 TCID_50_/ml/h [data are from (28) and they are indicated in the box above the panel, for mock, HCV p0, HCV p100 and HCV p200, respectively] and the relative H3Ser10ph densitometry values at time 72 hours post-infection (given in panel B). The discontinuous line corresponds to function y=-3.45x+0.81 (R^2 =^ 0.77, p-value=0.1227; linear regression test). **(D)** Same as B but for viral protein NS5A. **(E)** Same as C but for the correlation between viral protein NS5A and cellular protein H3Ser10ph levels (densitometry values in panels B and D). The discontinuous line corresponds to function y=-1.33x+1,13 (R^2 =^ 0.77; p-value=0.00018; linear regression test). **(F)** Same as B but for viral core protein. **(G)** Same as C but for the correlation between viral core protein and H3Ser10 levels (densitometry values in panels B and F). The discontinuous line corresponds to function y=-2.13x+1.12 (R^2 =^ 0.85; p-value=0.000018; linear regression test).

A previous study indicated that at early times after infection with HCV p0 [the virus with a basal fitness level (Perales et al., 2013)] resulted in a reduction of H3Ser10ph (Madejón et al., 2015). To investigate the weight of HCV fitness in this reduction, Huh-7.5 cells were either mock-infected or infected with HCV p0, HCV p100 and HCV p200 at a multiplicity of infection (MOI) of 0.03 TCID_50_/cell. H3Ser10ph levels were measured in cells lysed at 24h, 48h, and 72h post-infection, and compared with those of viral proteins NS5A and core as markers of the intracellular extent of the infection. Proteins were analysed by western blot assay using a specific polyclonal antibody for H3Ser10ph, a monoclonal antibody for NS5A, and a monoclonal antibody for core; β-actin, was used as loading control (see Materials and Methods for the origin of the antibodies). The results (**Figures 2A-C**) indicate that high HCV fitness accentuated the decrease of H3Ser10ph that reached a maximum of 5-fold with HCV p200 at late times post-infection; a reduction was observed at 48h post-infection with HCV p200, while a comparable effect required 72h with HCV p100 or HCV p0. The decrease correlated with an increase of intracellular infectivity and viral NS5A and core protein levels (**Figures 2D-G**). To reinforce the results, H3Ser10ph expression levels were compared between the low fitness HCV p0 population and the high fitness populations HCV p100 and HCV p200. The results showed again a significant decrease of H3Ser10ph levels comparing HCV p0 with HCV p100 and HCV p200 at late times post-infection (**Figure S1A**). Similar results were obtained for NS5A levels, while no significant differences were observed in core levels after infection with HCV p0 compared to HCV p200 (**Figures S1B, C**).

**Figure 3:**
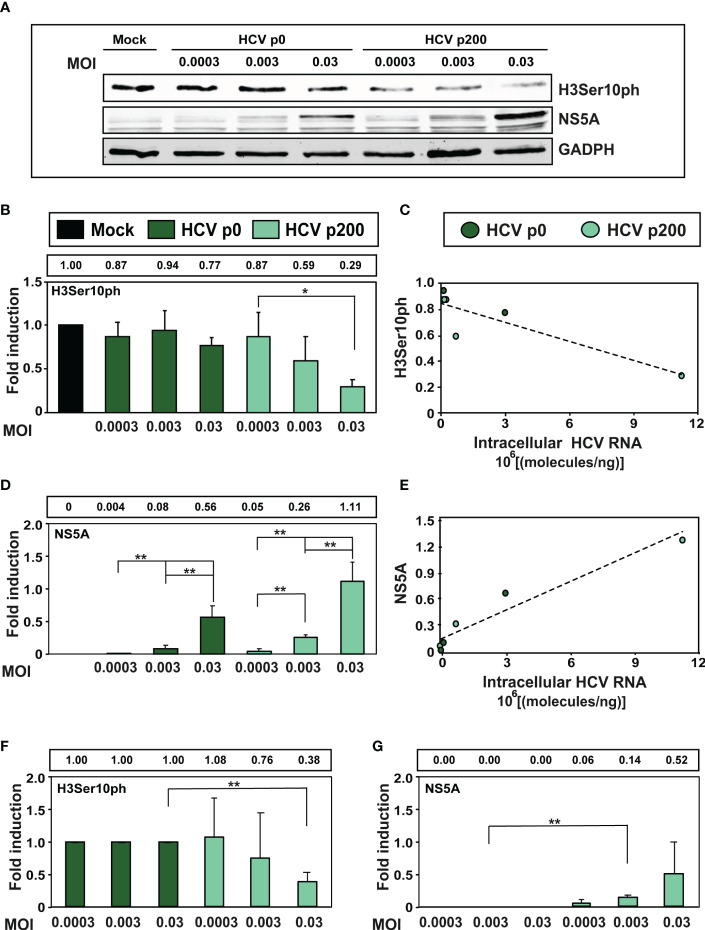
Effect of the multiplicity of infection (MOI) on the intracellular H3Ser10ph level. **(A)** Western blot analysis of extracts of cells that were either mock-infected or infected with HCV p0 or HCV p200 at the indicated MOI. Extracts were prepared at 72h post-infection. GAPDH was used as the loading control. **(B)** H3Ser10ph levels in mock-infected or HCV-infected cells, expressed as the fold induction relative to the corresponding value for the mock-infected cells. The values are the average (and standard deviations) of the densitometric quantifications of three independent western blots; the image of one of them is shown in **(A)** The MOI and infecting virus are indicated in abscissa, and the densitometry values (using as reference the corresponding value for the extract of mock-infected cells, taken as 1) are written in the box above the panel. **(C)** Correlation between the intracellular amount of viral RNA measured at 72h post-infection (abscissa) and the H3Ser10ph level determined by densitometry of the Western blots (ordinate). The discontinuous line corresponds to function y=-5x10^-0.8^x+0.85 (R^2 =^ 0.78; p-value=0.02027, linear regression test). **(D)** Same as B but for viral protein NS5A. **(E)** Same as C but for viral protein NS5A. The discontinuous line corresponds to function y=1x10^-0.7^x + 0.12 (R^2 =^ 0.918; p-value=0.0017, linear regression test). **(F, G)** Same as B and D but with the H3Ser10ph and NS5A levels in HCV-infected cells expressed as the fold induction relative to the corresponding value for the HCV p0 infected cells. The statistical significance of differences is as follows: *p<0.05; **p<0.01; unpaired t -test.

Since high viral fitness entails an increase of the intracellular level of viral RNA and its expression proteins, we examined the consequences for H3Ser10ph levels of infecting cells with HCV p0 and HCV p200 at MOIs that were up to 100-fold lower than that used in our standard infection protocol. The results (**Figure 3**) confirmed the effect of HCV fitness on H3Ser10ph depletion and its attenuation at low MOI that decreased the average level of intracellular NS5A. Similar results were obtained by comparing H3Ser10ph and NS5A levels between HCV p0 and HCV p200 (**Figures 3F, G**). Thus, HCV fitness is a determinant of the intracellular decrease of H3Ser10ph.

### The effect of HCV fitness on the intracellular level of Aurora kinase B

**Figure 4:**
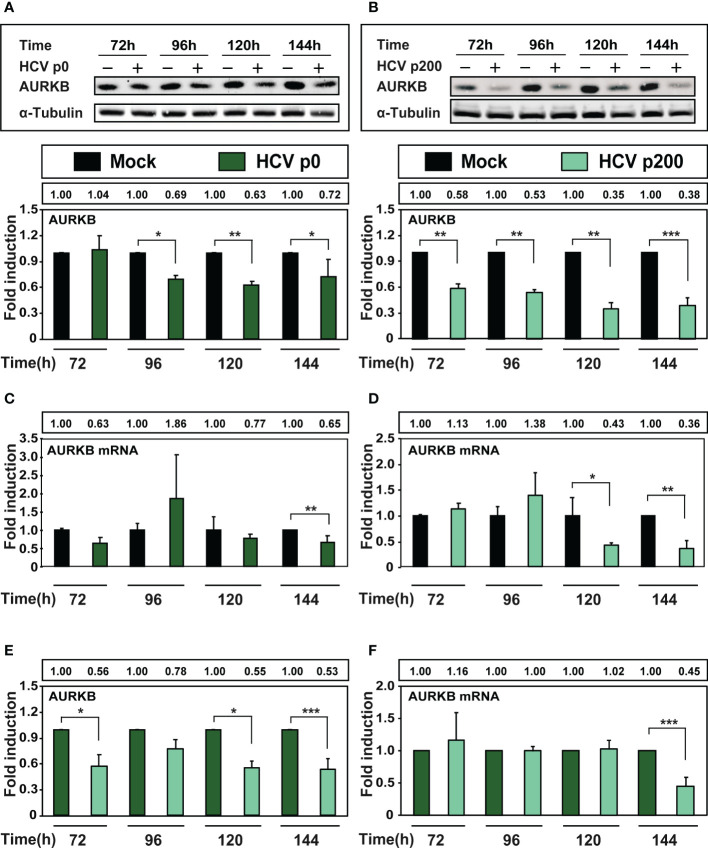
Effect of HCV infection on AURKB expression. Huh-7.5 reporter cells were either mock-infected or infected with HCV p0 and HCV p200 at a MOI of 0.03 TCID_50_/cell. **(A)** Western blot with extracts of cells infected with HCV p0. Bands correspond to the cellular AURKB and α-tubulin used as loading control. The lower panel shows the densitometry quantification of the bands, taking as reference the corresponding value for mock-infected cells, numerical values are given in the box above the lower panel. Times post-infection are indicated in abscissa. **(B)** Same as A but for HCV p200-infected cells. **(C, D)** RT-qPCR measurements of AURKB mRNA using GADPH gene as control. **(E, F)** Same as A-D but with the AURKB and AURKB mRNA levels in HCV-infected cells expressed as the fold induction relative to the corresponding value for the HCV p0 infected cells. Results are the average of three independent experiments (biological triplicate). Asterisks indicate statistical significance as follows: *p<0.05; **p<0.01; ***p<0.001; unpaired t-test.

AURKB is one of the enzymes that phosphorylates Ser10 of histone H3. Reduction of H3Ser10ph levels upon HCV infection is mediated by AURKB through its interaction with the viral core protein, as measured at early times post-infection (Madejón et al., 2015). To examine if HCV fitness affected the intracellular amount of AURKB, protein and intracellular RNA extracts were prepared from cells either mock-infected or infected with HCV p0 or HCV p200 at a MOI of 0.03 TCID_50_/cell. AURKB was detected by western blot using a specific polyclonal antibody. The results (**Figures 4A, B**) show significant reductions of AURKB at late times post-infection, that were more accentuated with HCV p200 than HCV p0; the difference between the two viruses reached 3.3-fold at 144 h post-infection. To examine if the decrease of AURKB proteins levels corresponded with a decrease in AURKB mRNA levels, RNA extracts were quantified by RT-qPCR. The results (**Figures 4C, D**) show a significant decrease in AURKB mRNA levels at late-times post-infection, and the decrease was enhanced with high fitness HCV p200. Therefore, a significant decrease was also observed by comparing ARUKB protein and mRNA levels between cells infected with HCV p0 or HCV p200 (**Figures 4E, F**). Therefore, AURKB kinase itself is affected by the replicative fitness of the infecting HCV.

### The influence of HCV fitness on other epigenetic markers

**Figure 5:**
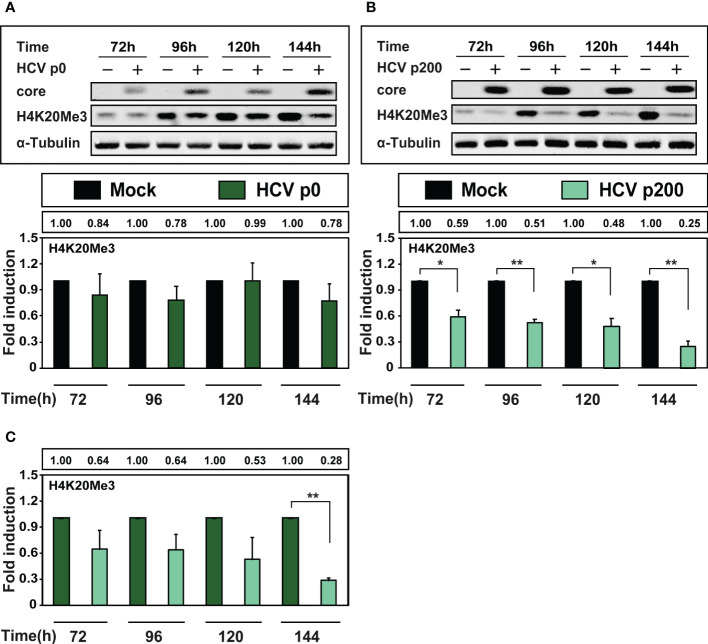
Comparison of the effect of HCV infection on the level of H4K20Me3. Huh-7.5 reporter cells were either mock-infected or infected with HCV p0 or HCV p200 at a MOI of 0.03 TCID_50_/cell, and cellular extracts were prepared at the indicated times post-infection. Viral core protein, cellular H4K20Me3, and α-tubulin (used as loading control) were visualized by Western blot. **(A)** Upper panel: Western blot analysis of cells infected with HCV p0; below, densitometry quantifications (bars) with numerical values written in the box above the panel; Times post-infection are indicated in abscissa. **(B)** Same as A but for HCV p200-infected cells. **(C)** Same as **(A, B)** but with the H4K20Me3 levels in HCV-infected cells expressed as the fold induction relative to the corresponding value for the HCV p0 infected cells. Results are the average of three independent experiments (biological triplicate). Asterisks indicate statistical significance as follows: *p<0.05; **p<0.01; unpaired t-test.

HCV infection results in variation of several epigenetic markers (Madejón et al., 2015; Lohmann and Bartenschlager, 2019; Perez et al., 2019; Zheng et al., 2019). A particularly relevant signature is the tri-methylated form of lysine 20 of histone H4 (H4K20Me3) since its depletion is a common finding among tumor cells (Fraga et al., 2005). Therefore, we measured H4K20Me3 in extracts of cells at late times after infection with HCV p0 or HCV p200 at a MOI of 0.03 TCID_50_/cell, by western blot using a specific polyclonal antibody against H4K20Me3 (**Figure 5**). Interestingly, there was only a minor (but not statistically significant) decrease of H4K20Me3 upon infection with HCV p0, but a highly significant decrease at all times post-infection with HCV p200, that reached 4-fold at 144h post-infection (**Figure 5**). In addition, a significant decrease was also observed at late time post-infection comparing H4K20Me3 levels between cells infected with HCV p0 or HCV p200 (**Figure 5C**). Therefore, and most significant for the effect of HCV fitness on epigenetic signatures, depletion of H4K20Me3 was noted only upon infection with high fitness HCV.

## Discussion

In the present investigation we have explored the effect of HCV fitness on several epigenetic markers at late times post-infection of Huh-7.5 cells, when the great majority of cells in the culture are infected, and the alterations of host gene expression are fully manifested (Moreno et al., 2017). The study has revealed two categories of fitness influence: (i) markers that are altered by HCV p0 (the population displaying basal fitness), with a significant accentuation when the cells are infected with high fitness HCV p200 (i.e. effects on H3Ser10ph and AURKB); (ii) a marker whose intensity was modified only in infections with high fitness HCV p200 (H4K20Me3). This distinction is circumscribed by the 2.3-fold fitness range that can be reached with our experimental system (Sheldon et al., 2014; Moreno et al., 2017). Additional differences might be revealed if HCV displaying larger fitness differences were compared.

One of the most frequent strategies used by some viruses to dysregulate host gene expression involve epigenetic mechanisms. This is true also of many plant viruses (Wang et al., 2019), as part of modulation of gene expression in infected plant cells. In the case of tobacco etch potyvirus, Elena and colleagues documented that viral fitness had a clear influence on the host transcriptome profile (Cervera et al., 2018). Regarding animal viruses, epigenetic changes evoked by hepatitis B virus (HBV) infection affected both viral cccDNA and host DNA expression, with implications for HBV-associated HCC (Levrero and Zucman-Rossi, 2016; Dandri, 2020). Epigenetic mechanisms probably contributed to suppression of the IFN response during rabies virus infection of mice (Abdulazeez et al., 2020). Histone modifications affected herpes simplex virus 1 (HSV-1) gene expression in THP-1 cells (Gao et al., 2020). Viral respiratory infections, particularly by rhinovirus and respiratory syncytial virus, may participate in the exacerbation of airway inflammatory disease through DNA methylation and histone modifications of cells from the airway epithelium (Caixia et al., 2019; Tan et al., 2020). Likewise, modifications of epigenetic signatures have been associated with arbovirus infections (de Aguiar et al., 2019). In the studies with several DNA and RNA animal viral pathogens, fitness was not taken into consideration, and its possible relevance in the interpretation of types and extent of epigenetic modifications remains unknown.

Our study has established fitness as a relevant parameter with regard to the occurrence and intensity of epigenetic modifications underwent by the host cells. The results are in line with the fact that such modifications are often dependent on the amount of intracellular viral proteins that interact with cellular proteins, and high fitness virus provide a larger supply of interacting proteins (Sheldon et al., 2014; Moreno et al., 2017). Viral fitness may be also relevant to epigenetic therapeutic approaches (Ramos and Lossos, 2011; Dandri, 2020) because they target proteins that produce or recognize epigenetic marks, whose extent may be dependent on fitness of the effector virus. Thus, there is a variety of scenarios in which the types of influences revealed by our study may be pertinent.

A limitation of our study is that we cannot define in a precise way the molecular mechanism which is responsible of the observed fitness effects. Despite HCV p0 and HCV p200 belonging to the same evolutionary lineage, the mutant spectrum composition and the dominant sequences differ considerably between the two populations (Gallego et al., 2020; Delgado et al., 2021). Therefore, the repertoire of genomes and their expression products that intracellularly may act on the epigenetic mark effectors differ between HCV p0 and HCV p200. A second, non-mutually exclusive influence may be exerted by the number of Huh-7.5 cells that were infected at the times when the epigenetic marks were measured, following the initial infections at a MOI of 0.03 TCID_50_/cell (see Materials and Methods).

Measurement of the number of infected cells using the anti-NS5A monoclonal antibody 9E10 indicated a significantly larger percentage of infected cells with HCV p200 than HCV p0, although the difference was not significant at 144 h post-infection (**Figure S2**). Yet a third non-mutually exclusive influence is the intracellular viral load which was quantified as being 2 to 3 logarithms larger for HCV p200 than HCV p0 at early (up to 21 h) and late (up to 72 h) post-infection (Moreno et al., 2017). Viral fitness is a parameter that captures several features of virus-host interactions (Domingo and Holland, 1997; Domingo et al., 2019), and the three effects outlined here may contribute to triggering epigenetic differences among host cells. A limitation of this work is the use of a tumorigenic human hepatoma cell line because this is the cell line in which HCV with higher fitness was obtained. However, similar results were obtained after the expression of HCV core in human primary hepatocytes (Madejón et al., 2015).

Persisting epigenetic changes following HCV infection may lead to HCC, and its recurrence in some patients with advanced fibrosis (Hamdane et al., 2019). Of the epigenetic signatures that were modified due to HCV fitness, AURKB and H4K20Me3 are especially noteworthy for their implication in cancer. AURKB is the effector of H3Ser10 phosphorylation (Goto et al., 2002), and the decrease of both H3Ser10ph and of AURKB was HCV fitness-dependent. AURKB is altered in several types of tumor cells, including HCC (Tanaka et al., 2008; Lin et al., 2010; Zhou et al., 2018). This kinase has been suggested as an independent molecular marker predicting tumor invasion of HCC (Tanaka et al., 2008). Previous report from our group indicated that the inhibition of AURKB could be one of the mechanisms by which HCV decreases cell proliferation and viability (Madejón et al., 2015). A striking case was a remarkable 4-fold depletion of H4K20Me3 produced by high fitness HCV and not by the basal fitness HCV population. This marker is involved in repression of transcription and genomic instability, an established specific process related to cancer development (Wang et al., 2008) and gene silencing (Schotta et al., 2004). Its depletion is common among tumor cells (Fraga et al., 2005), and it has been also reported among the epigenetic alterations associated with nonalcoholic steatohepatitis-related HCC in Stelic animal model mice, a system in which the course of disease resembles that of humans (de Conti et al., 2017). It is remarkable that in our cell culture system the decrease of H4K20Me3 was only seen with high fitness virus.

Our study establishes HCV fitness as a relevant parameter for epigenetic modifications and raises several questions which are presently under study. One is the duration of the epigenetic modifications observed once the virus is no longer present in the hepatic cell, and whether the fitness-dependent alterations are reverted. To solve this question poses the challenge that high fitness HCV cannot be extinguished by treatment with anyone of the HCV inhibitors that we have previously tested individually (Gallego et al., 2016; Gallego et al., 2018), and that the antiviral agents themselves can affect epigenetic marks (Giovannini et al., 2020). Our recent studies on HCV population dynamics in cell culture have documented that fitness gain was accompanied of a broadening of mutant spectra composed of many co-dominant genomes with comparable fitness (Domingo et al., 2020; Gallego et al., 2020). If these observations in population dynamics applied to HCV replication in the liver, they would suggest that chronicity may favor HCV fitness increase, with its concomitant elevated probability of epigenetic perturbations conductive to HCC. Fitness of the HCV that initially infects a patient may also have an effect on HCC development. However, comparative measurements of HCV fitness *in vivo* are presently not attainable, and the same virus may display dissimilar fitness in different host individuals.

## Data availability statement

The raw data supporting the conclusions of this article will be made available by the authors, without undue reservation.

## Author contributions

AS-P, AM, JG-S, ED and CP conceived, designed, and supervised the project. CG-C, IF-R, IG, MC, MS, AL, AA performed the experiments, analyzed the results and performed the statistical analyses. AM, JG-S, ED, AS-P and CP wrote the manuscript. All authors contributed to the article and approved the submitted version.

## Supplementary material

Supplementary Figure 1 Effect of HCV fitness on the level of cellular protein H3Ser10ph and viral proteins NS5A and core. Huh-7.5 reporter cells were either mock-infected or infected with HCV p0, HCV p100 or HCV p200 (abbreviated as p0, p100 and p200, respectively) at an initial MOI of 0.03 TCID50/cell; protein extracts were prepared at the indicated times post-infection. **(A)** H3Ser10ph levels in HCV-infected cells expressed as the fold induction relative to the corresponding value for the HCV p0 infected cells; the infecting HCV (code in bottom right part), and the time post-infection at which extracts were prepared are given in the abscissa; the numerical densitometry values (measured relative to the HCV p0-infected sample, taken as 1) are indicated in the upper box, next to the panel. The values are the result of three independent experiments (biological triplicate). **(B)** Same as A but for viral protein NS5A. **(C)** Same as B but for the viral protein core. Asterisks indicate statistical significance as follows: *=p.

Supplementary Figure 2 Percentage of infected cells upon infection with HCV p0 and HCV p200. Cells were infected with the viruses HCV p0 or HCV p200 at a MOI of 0.03 TCID50/cell and were fixed with methanol at a different times post-infection. Cells were stained to detect NS5A using anti-NS5A monoclonal antibody 9E10. **(A)** Images of the stained cells at a different times post-infection. The images were taken using the LSM900 Upright Confocal Microscope. The different times post-infection are indicated in the upper boxes. The infecting viruses are indicated in the right boxes. **(B)** Percentage of infected cells at a different times post-infections. The number of infected cells was calculated using the software “Image Fiji”. The infecting virus is indicated in the upper box. The percentage of infected cells is indicated in the ordinate while the time post-infection is indicated in the abscissa. Numerical values are given in the box above the lower panel. Asterisks indicate statistical significance as follows: *** =p.
