# Supplementary material for: Hepatitis C virus fitness can influence the extent of infection-mediated epigenetic modifications in the host cells
Source: Front Cell Infect Microbiol. 2023 Mar 13;13:1057082. doi: 10.3389/fcimb.2023.1057082 (PMC10040758; doi:10.3389/fcimb.2023.1057082)
Supplement: Supplementary Figure 1 — Effect of HCV fitness on the level of cellular protein H3Ser10ph and viral proteins NS5A and core. Huh-7.5 reporter cells were either mock-infected or infected with HCV p0, HCV p100 or HCV p200 (abbreviated as p0, p100 and p200, respectively) at an initial MOI of 0.03 TCID50/cell; protein extracts were prepared at the indicated times post-infection. (A) H3Ser10ph levels in HCV-infected cells expressed as the fold induction relative to the corresponding value for the HCV p0 infected cells; the infecting HCV (code in bottom right part), and the time post-infection at which extracts were prepared are given in the abscissa; the numerical densitometry values (measured relative to the HCV p0-infected sample, taken as 1) are indicated in the upper box, next to the panel. The values are the result of three independent experiments (biological triplicate). (B) Same as A but for viral protein NS5A. (C) Same as B but for the viral protein core. Asterisks indicate statistical significance as follows: *=p. [file DataSheet_1.pdf]

<u>Supplemental material</u>:

Hepatitis C virus fitness can influence the extent of infection-mediated epigenetic modifications in the host cells

Carlos García-Crespo[a#], Irene Francisco-Recuero[b#], Isabel Gallego[a,c], Marina Camblor[b], María Eugenia Soria[a,d], Ana López[b], Ana Isabel de Ávila[a], Antonio Madejón[c,e], Javier García-Samaniego[c,e], Esteban Domingo[a,c*], Aurora Sánchez-Pacheco[b*] and Celia Perales[c,d,f*]

[a]*Department of Interactions with the Environment, Centro de Biología Molecular "Severo Ochoa" (CSIC-UAM), Consejo Superior de Investigaciones Científicas (CSIC), Campus de Cantoblanco, 28049, Madrid, Spain*

[b]*Department de Biochemistry, UAM, Instituto de Investigaciones Biomédicas Alberto Sols, CSIC-UAM, Arturo Duperier 4, 28029 Madrid, Spain*

[c]*Centro de Investigación Biomédica en Red de Enfermedades Hepáticas y Digestivas (CIBERehd), Instituto de Salud Carlos III, 28029 Madrid, Spain*

[d]*Department of Clinical Microbiology, IIS-Fundación Jiménez Díaz, UAM. Av. Reyes Católicos 2, 28040 Madrid, Spain*

[e]*Hepatology Unit Hospital Universitario La Paz/Carlos III. Instituto de Investigación Sanitaria "La Paz", Madrid, Spain*

[f]*Department of Molecular and Cell Biology, Centro Nacional de Biotecnología (CNB-CSIC), Consejo Superior de Investigaciones Científicas (CSIC), Campus de Cantoblanco, 28049 Madrid, Spain*

#Both authors have contributed equally to this work; names written in inverse alphabetical order

*Corresponding authors: Esteban Domingo (edomingo@cbm.csic.es), Aurora Sánchez-Pacheco (asanchez@iib.uam.es) and Celia Perales (celia.perales@cnb.csic.es)

Figure S1

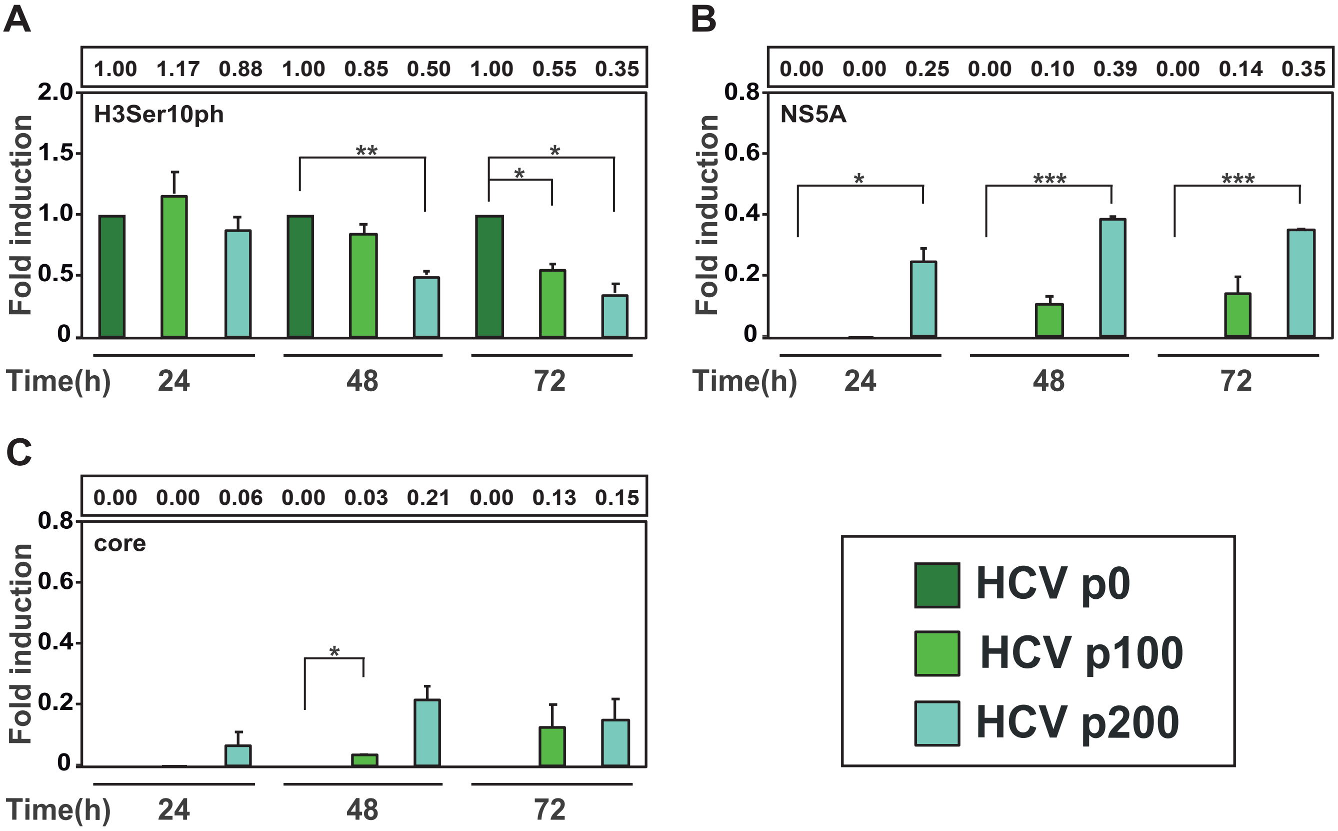

Figure S1. Effect of HCV fitness on the level of cellular protein H3Ser10ph and viral proteins NS5A and core. Huh-7.5 reporter cells were either mock-infected or infected with HCV p0, HCV p100 or HCV p200 (abbreviated as p0, p100 and p200, respectively) at an initial MOI of 0.03 TCID50 / cell; protein extracts were prepared at the indicated times post-infection. **(A)** H3Ser10ph levels in HCV-infected cells expressed as the fold induction relative to the corresponding value for the HCV p0 infected cells; the infecting HCV (code in bottom right part), and the time post-infection at which extracts were prepared are given in the abscissa; the numerical densitometry values (measured relative to the HCV p0-infected sample, taken as 1) are indicated in the upper box, next to the panel. The values are the result of three independent experiments (biological triplicate). **(B)** Same as A but for viral protein NS5A. **(C)** Same as B but for the viral protein core. Asterisks indicate statistical significance as follows: *=p<0.05; **=p<0,01; ***=p<0.00; unpaired t-test.

Figure S2

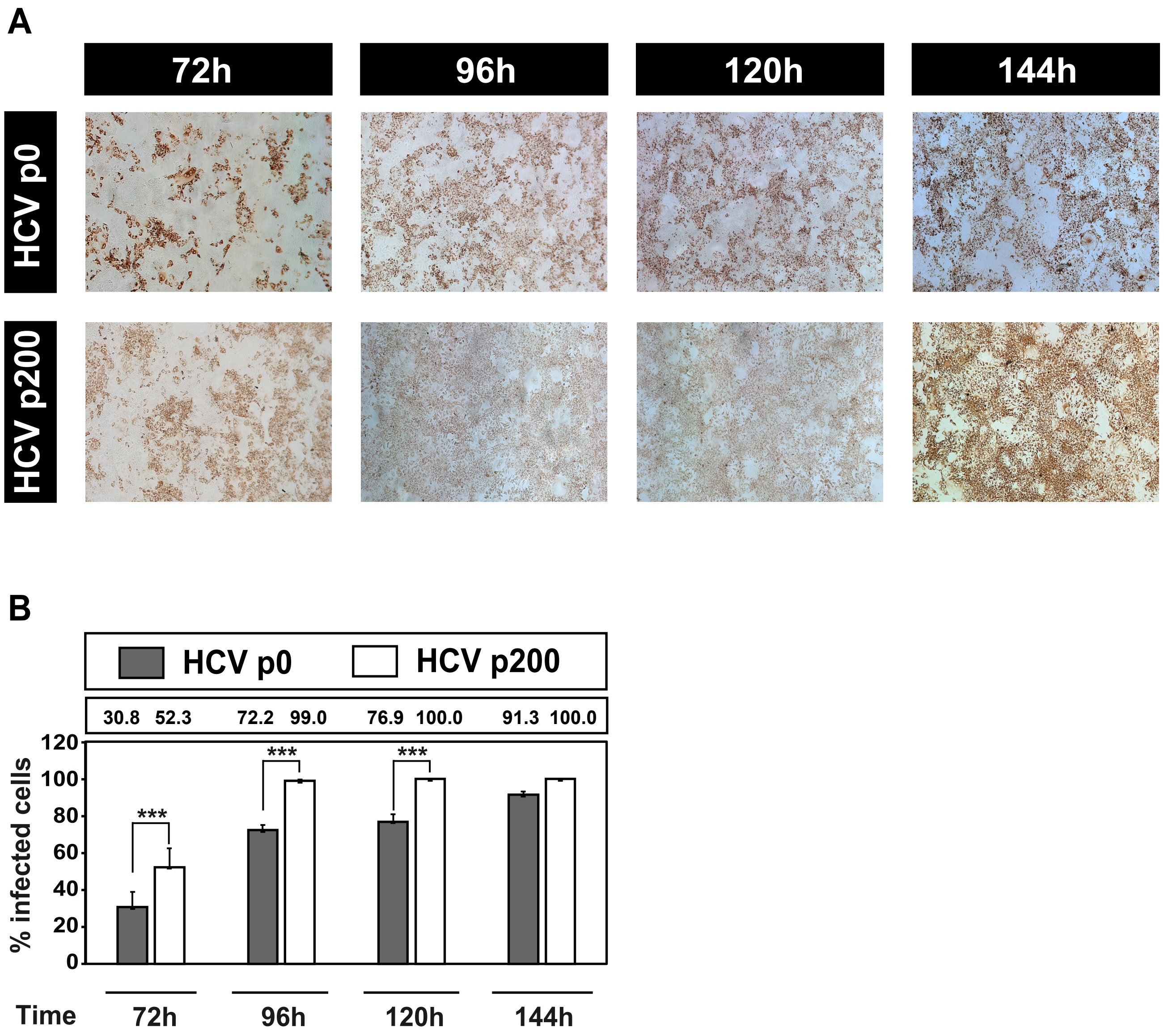

Figure S2. Percentage of infected cells upon infection with HCV p0 and HCV p200. Cells were infected with the viruses HCV p0 or HCV p200 at a MOI of 0.03 TCID50/cell and were fixed with methanol at a different times post-infection. Cells were stained to detect NS5A using anti-NS5A monoclonal antibody 9E10. **(A)** Images of the stained cells at a different times post-infection. The images were taken using the LSM900 Upright Confocal Microscope. The different times post-infection are indicated in the upper boxes. The infecting viruses are indicated in the right boxes. **(B)** Percentage of infected cells at a different times post-infections. The number of infected cells was calculated using the software "Image Fiji". The infecting virus is indicated in the upper box. The percentage of infected cells is indicated in the ordinate while the time post-infection is indicated in the abscissa. Numerical values are given in the box above the lower panel. Asterisks indicate statistical significance as follows: *** =$p<0.001$; proportion test.
